# A survey of implicit bias training in physician assistant and nurse practitioner postgraduate fellowship/residency programs

**DOI:** 10.1186/s12909-022-03664-5

**Published:** 2022-08-03

**Authors:** Vasco Deon Kidd, Jennifer M. Spisak, Sarah Vanderlinden, Gerald Kayingo

**Affiliations:** 1 School of Medicine, Department of Orthopaedic Surgery, University of California Irvine (UCI Health), 101 The City Dr S, Orange, CA 92868 USA; 2 Ronald O. Perelman Department of Emergency Medicine, NYU Langone Health, 545 First Avenue, Greenberg Hall Suite 6B, New York, NY 10016 USA; 3 Department of Surgery, Trauma and Critical Care, Medical College of Wisconsin, Milwaukee, WI USA; 4 Graduate School, University of Maryland Baltimore, Baltimore, MD USA

**Keywords:** Implicit bias, Unconscious bias, Diversity training, Postgraduate education, Fellowship, Residency, Physician assistant, Physician associate, Nurse practitioner, Advanced practice provider

## Abstract

**Background:**

There has been renewed focus on advancing inclusivity within organized medicine to reduce health disparities and achieve health equity by addressing the deleterious effects of implicit bias in healthcare and clinical outcomes. It is well documented that negative implicit attitudes and stereotypes perpetuate inequity in healthcare.

The aim of this study is to investigate implicit bias training in postgraduate physician assistant (PA) and nurse practitioner (NP) education; describe delivery of content to trainees; and detail program directors’ attitudes toward this type of training. Although there is research examining implicit bias training in physician residency education, there are no published studies on implicit bias training in postgraduate PA and NP postgraduate residency/fellowship programs.

**Method:**

A non-experimental, descriptive study was designed to obtain information via survey from members of the Association of Postgraduate Physician Assistant Programs (APPAP).

**Results:**

The response rate was 41%. The majority of respondents (76%) felt that PA and NP postgraduate programs should include implicit bias instruction. Educational strategies used by PA and joint PA/NP postgraduate programs or their sponsoring institution to deliver implicit bias content to trainees include: implicit bias training modules (50%), facilitated group discussions (36%), invited speaker on implicit bias (33%), case studies on implicit bias (16%), and implicit association test (10%); however, 30% of postgraduate programs do not provide implicit bias training to PA and/or NP trainees. Barriers to implementing implicit bias training expressed by some postgraduate programs include: uncertainty in how to incorporate implicit bias training (16%); lack of strategic alignment with training program or sponsoring institution (13%); time constraints (10%); financial constraints (6%); lack of access to content experts (6%); and unfamiliarity with evidence supporting implicit bias training (6%).

**Conclusion:**

The present study sheds some light on the current state of implicit bias training in PA and joint PA/NP postgraduate residency/fellowship programs. While the majority of programs offer some sort of implicit bias training, there is a need to standardize this training in PA and joint PA/NP postgraduate education curricula using an actionable framework.

## Background

A recent wave of protests triggered by perceived racial injustice and systemic inequalities in American society has placed a spotlight on the importance of addressing these pervasive challenges through multi-faceted diversity, equity, and inclusion (DEI) initiatives. One such approach to address longstanding grievances has been to expand implicit bias training across all sectors of society. Implicit bias is defined as unconscious and/or automatic mental associations made between the members of a social group (or individuals who share a particular characteristic) and one or more attributes (implicit stereotype) [1]. In healthcare, implicit bias training began to take shape after the release of the 2003 Institute of Medicine (IoM) report entitled, Unequal Treatment: Confronting Racial and Ethnic Disparities in Health Care, which highlighted structural health inequalities among racial and ethnic minorities [2]. Moreover, the report acknowledged the role of implicit bias in exacerbating health outcomes. This has led to a national call for cementing implicit bias training strategies for all healthcare professionals as the country strives to increase awareness of subconscious beliefs or attitudes and their impact on clinical outcomes.

Previous research has shown that healthcare professionals exhibit the same levels of implicit bias as the wider population, which can lead to poor quality care [3]. For example, health care providers appear to have positive attitudes toward whites and negative attitudes toward people of color [3–6]; it follows that minorities lag behind the white population in preventive screening rates as well as access to specific medical interventions [1]. This may be due in part to a lack of social consensus about the role of automatically-activated associations in influencing provider behaviors and the need for strategies to adjust automatic patterns of thinking. Consequently, implicit bias has been implicated in adverse patient-clinician interactions, including medical decision-making [3, 7–9]. Research with resident physicians has shown that biases of medical educators, can negatively influence trainee education as they model their educators' behaviors and actions [10]. Also, repetitive experiences of racial bias experienced by residents have been linked to burnout and mental health issues [11, 12]; Therefore, creating and cultivating an inclusive and culturally competent health care workforce is critical in addressing patients and trainees with diverse backgrounds, values, beliefs, and ways of thinking [13].

Existing efforts to expand and support implicit bias training have become a priority for physician residencies over the course of their training [14–24]. PA postgraduate residency training began in 1970s and NP postgraduate residency training in 2007. PA and joint PA/NP postgraduate residency/fellowship programs offer abbreviated specialty training in a variety of medical and surgical specialties (12 months or longer), but are not required for initial certification or state licensure. There are several pathways available for NP postgraduate programs to obtain accreditation and PA postgraduate programs can earn accreditation under a newly developed pathway with updated standards. Nonetheless, a portion of postgraduate programs have secured accreditation, which remains a voluntary process. The Accreditation Review Commission on Education for the Physician Assistant (ARC-PA) and The National Nurse Practitioner Residency and Fellowship Training Consortium have adopted standards linked to diversity in postgraduate education. For example, the ARC-PA offers the following standards (http://www.arc-pa.org/postgraduate-accreditation/).

- **B1.11-** The curriculum must include instruction to prepare the PA trainee to provide medical care to patients from diverse populations.
- **B1.12**- The curriculum must include instruction that addresses disparities in the health status of people from diverse racial, ethnic, and culture background.

However, it remains unclear whether implicit bias training is being offered in PA and joint PA/NP postgraduate residency/fellowship education and if not, what are the perceived barriers that exist in delivering this content.

It should be noted that there is ample research indicating that PAs and NPs provide excellent clinical care without postgraduate residency/fellowship training [25, 26]; nevertheless, there has been an expanded interest in these postgraduate programs from both federal and public sectors due in part to projected shortfalls in physician specialties and a desire among some PAs and NPs to improve “clinical readiness” through transition-to-practice opportunities. Additionally, some academic health systems have adopted PA and joint PA/NP fellowship/residency training programs to bolster recruitment and retention strategies of qualified career staff [27]. In the last few years, there has also been a consistent trend towards increased growth of research in the field of postgraduate PA and NP training [27–34], though a dearth of published studies investigating diversity efforts in these programs remains.

Hence, the overarching aim of this study is to identify potential barriers, attitudes, and strategies about implicit bias training in postgraduate PA and joint PA/NP member programs affiliated with the Association of Postgraduate PA Programs (APPAP). This organization was founded in 1988 to expand specialty education for PAs and serves as a resource for existing programs while advising institutions interested in developing postgraduate educational programs for various medical and surgical disciplines in the United States. Organizational membership is approved based on meeting membership criteria and includes representation from diverse specialty programs across the United States. The organization collaborates with the Association of Postgraduate APRN programs (APGAP) and includes members with joint membership to represent joint PA/NP postgraduate platforms.

## Method

A non-experimental, descriptive research study was designed to obtain information from postgraduate programs affiliated with APPAP membership. After review of the implicit bias literature, a web-based survey was developed and consists of 18 items. Four experts with content expertise reviewed the survey items. The content experts involved in the pilot did not participate in the overall survey. Individual APPAP member postgraduate programs were sent an email invitation with a link to a voluntary, anonymous, online survey. The email introduction to the survey contained all the necessary elements of written consent and submission of the survey indicated the respondents’ consent to participate.

The survey was distributed by the APPAP administrator/membership manager to postgraduate programs affiliated with APPAP membership. The study period was from January 5, 2022 through February 5, 2022. Six email reminders were sent to non-respondents over the study period to ensure the highest possible response rate. The participants completed the survey through a secure, commercially available internet platform (SurveyMonkey), and confidentiality was maintained throughout the study. The average length of time to complete the survey was 3 min. No identifying information was obtained. Survey responses were aggregated, and descriptive statistical analyses were conducted using the statistical package embedded within the survey software. While the majority of survey questions 16 (88%) were closed-ended, study participants were asked their opinions on four implicit bias survey questions and responses were evaluated on a five-point Likert scale (ranging from 1, strongly disagree, to 5, strongly agree). In our data analysis, we decided to aggregate the positive ratings “strongly agree” and “agree” and negative ratings “strongly disagree” and “disagree” to draw meaningful conclusions. When calculating sample size, we estimated that 39 (53%) or more survey responses were needed to have a confidence level of 95% within a 10% margin of error. The survey is exempt research confirmed by the University of California Irvine Institution Review Board and the protocol was approved on January 3, 2022.

## Results

Among the 73 invited postgraduate programs, 30 completed the entire survey. Two programs submitted incomplete surveys with less than half the total items completed, were excluded from data analysis. The final response rate is (41%). Response rates varied by specialty: emergency medicine 8 (26%), general surgery 3 (10%), psychiatry 3 (10%), multispecialty 2 (6%), family medicine 2 (6%), combined internal medicine and hematology/oncology 1 (3%), cardiovascular surgery 1 (3%), pediatric surgery and pediatric orthopaedic surgery 1 (3%), pediatric emergency medicine 1 (3%), hematology/oncology 1 (3%), cardiothoracic surgery 1 (3%), critical care medicine 1 (3%), medical oncology 1 (3%), hospitalist 1 (3%), acute care surgery 1 (3%), otolaryngology 1 (3%), other 1 (3%). The majority of respondents to the survey were PAs. Of those that completed the survey, 21 (70%) were program directors, 3 (10%) associate program directors, 2 (6%) medical directors, 2 (6%) academic/administrative coordinators, 1 (3%) advanced practice director, and 1 (3%) program manager. Sixty-three percent of respondents had at least four or more years of leadership experience and 27 (90%) of all respondents reported receiving implicit bias training within the last 3 years.

### Postgraduate program demographics

The 30 programs that completed the survey were organized either as single-track or multitrack programs. Thirty-six percent of respondents enroll both PAs and NPs, whereas 19 (63%) enroll PAs only. Moreover, 22 (73%) of respondents are located at an academic medical center, 6 (20%) community health center, 1 (3%) community hospital in a network and 1 (3%) hospital. The distribution of respondent programs among each census region was well represented except for the East south-central region, which has the lowest postgraduate program density (Table 1).

**Table 1:** Demographic distribution of program respondents

United States Region	Percent of Respondents
Middle Atlantic	26.6%
East North Central	13.3%
Mountain	13.3%
Pacific	10%
South Atlantic	10%
West North Central	10%
West South Central	10%
New England	6.6%
East South Central	0%

### Respondent perceptions toward implicit bias training

**Fig. 1:**
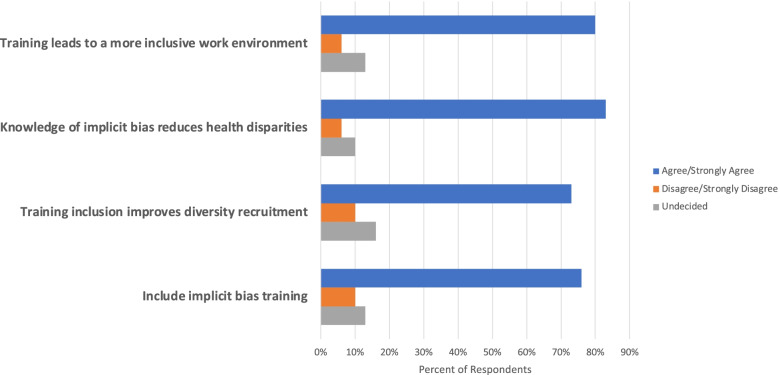
Respondent perceptions toward implicit bias training in PA/NP postgraduate education. *N* = 30

To explore perceptions regarding implicit bias training, a series of questions were asked. When asked about whether implicit bias training can lead to a more inclusive work environment for health care professionals, (80%) of respondents “strongly agree/agree” while (6%) “strongly disagree/disagree” and (13%) were undecided. Additionally, (83%) indicated “strongly agree/agree” that enhanced knowledge of implicit bias for healthcare professionals can help reduce healthcare disparities while (6%) “strongly disagree/disagree” and (10%) were undecided. Moreover, (73%) “strongly agree/agree” that implicit bias training in PA and joint PA/NP postgraduate education would help in the recruitment and selection of individuals from underrepresented backgrounds while (10%) “strongly disagree/disagree” and (16%) were undecided. Lastly, (76%) “strongly agree/agree” that PA and joint PA/NP postgraduate training should include implicit bias instruction while (10%) “strongly disagree/disagree” and (13%) were undecided. Our study findings demonstrate that the majority of respondents reported more favorable attitudes toward implicit bias training in PA and joint PA/NP postgraduate fellowship/residency training (Fig. 1).

Selected comments from respondents about implicit bias training

“*It [implicit bias training] is part of a comprehensive portfolio of professional identity and development.”*

*“It [implicit bias training] has been a great addition to our program, and we are incorporating it into orientation as well as throughout the year.”*

“*We have DEI faculty hired into our department.”*

*“We appreciate this survey and agree with integration of implicit bias training and APP postgraduate education.”*

### Educational strategies used in the delivery of implicit bias training

**Fig. 2:**
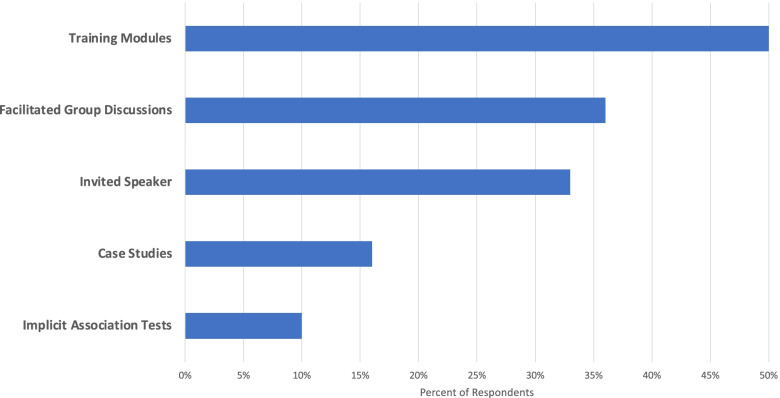
Implicit bias educational strategies in PA and NP postgraduate training program. *N* = 30. Respondents were permitted multi-select response

Through a series of questions, we probed what types of educational strategies are used by postgraduate residency/fellowship programs and/or the sponsoring institution to deliver implicit bias content and training to PA and NP trainees. We found that trainees are exposed to various forms of implicit bias training, including: implicit bias training modules (50%), facilitated group discussions (36%), invited speakers on implicit bias (33%), case studies on implicit bias (16%), and implicit association tests (IAT) (10%) (Fig. 2)**.** Most importantly, forty-three percent of postgraduate programs or their sponsoring institutions offer two or more educational strategies in providing implicit bias training to PA and NP trainees**.** Forty-six percent of postgraduate programs reported that implicit bias training was mandatory and (23%) indicated it was voluntary.

### Administration of implicit bias training

Postgraduate programs reported that implicit bias training is administered to their PA and NP trainees through the office for DEI (43%), department of human resources (40%), continuing education department (26%), or the postgraduate program (13%), teaching academy (3%), center for physician /advanced practice provider (MD/APP) leadership and development (3%), and physician residency program within same department (3%).

### Barriers to offering implicit bias training

**Fig. 3:**
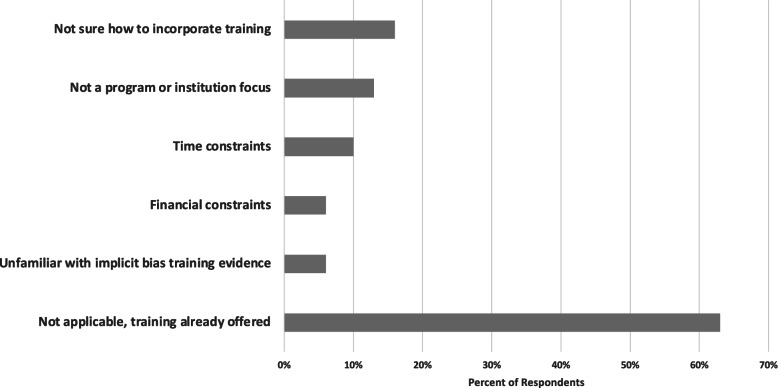
Barriers to offering implicit bias training in PA/NP postgraduate education. *N* = 30. Respondents were permitted multi-select response

Thirty percent of postgraduate program respondents do not offer implicit bias training to their PA and/or NP postgraduate trainees. The key barriers identified by postgraduate programs in not offering implicit bias training to postgraduate PA and NP trainees included: indecision as to how to incorporate implicit bias training in postgraduate training (16%), lacking strategic focus of the postgraduate program or sponsoring institution (13%), time constraints (10%), financial constraints (6%), and unfamiliarity with the evidence associated with implicit bias training (6%). Additionally, (16%) of programs were “unsure” if implicit bias training would be offered in the future to their PA and NP postgraduate trainees (Fig. 3).

## Discussion

There have been numerous studies that have assessed implicit bias training in graduate medical education (GME) focusing primarily on individual programs at a single institution. However, our novel cross-sectional study assessed the prevalence of implicit bias training as well as program directors’ attitudes toward this type of training across multiple postgraduate residency/fellowship programs at the national level. Our study findings demonstrate that the majority of respondents agree that implicit bias training can lead to a more inclusive work environment for health care professionals; reduce healthcare disparities; improve recruitment and selection of individuals from underrepresented backgrounds; and should be included as a component of PA and joint PA/NP postgraduate education curriculum. However, in some cases, certain barriers exist that prevent programs from incorporating and adapting implicit bias training such as time and financial constraints, misalignment of strategic focus, and unfamiliarly with the evidence regarding implicit bias training. Therefore, the results can serve to raise awareness among PA and joint PA/NP postgraduate faculty about specific opportunities and challenges in delivering implicit bias training content in their programs. The effectiveness of educational approaches in delivering implicit bias training content appears quite broad across the published literature.

For that reason, we recommend a focused framework for implicit bias training for PA and joint PA/NP postgraduate education previously described by Sukhera and Watling. The researchers outline a conceptual framework based on six key features for integrating implicit bias recognition into health professions education [35]. While this framework is comprehensive, it is best to narrow the focus to one of the six key features given the shorter training duration available in PA and joint PA/NP postgraduate education. After creating a safe, respectful, and inclusive learning environment to discuss implicit bias, we suggest a focus on emphasizing how implicit bias influences behaviors and patient outcomes. “For certain health professions, specific reference to literature on clinical decision making and cognitive psychology, including certain types of bias, such as anchoring (relying too heavily on the first piece of information about a patient) or confirmation bias (the tendency to favor information in a manner that confirms preexisting beliefs), may lay the groundwork for learners to engage with ideas about how biases may adversely affect care” [35].

The implicit association test (IAT) is a widely available tool that can be useful to incorporate into a focused framework for integrating implicit bias recognition into health professions education. The IAT was developed by Dr Anthony Greenwald in 1995 to measure the strength of associations between concepts and evaluations/stereotypes and has since become the worldwide standard for assessing implicit bias. Along with a team of scientists, he developed a non-profit organization, Project Implicit, that houses 15 free assessments (https://implicit.harvard.edu/implicit/research/). The IAT is not without its limitations; researchers have challenged its test/retest reliability, and ability to distinguish between cultural associations and personal preferences, and singular versus more beneficial assessment of multi-dimensional associations [36]. Nevertheless, the IAT can serve as a starting point to increase self-awareness of implicit bias and stand as a platform for the development of meaningful conversation amongst learners.

Furthermore, implicit bias training resources are widely available and accessible through membership in PA and NP sponsoring associations. The American Academy of PAs (AAPA) features a DEI Resource Center on their website; the resources available include Continuing Medical Education (CME), webinars, and podcasts; links to constituent organizations that are sponsoring ongoing DEI efforts as well as partner organizations who collaborate directly with AAPA in support of inclusivity; and links to other external resources, including articles, books, podcasts, videos, and webinars (https://www.aapa.org/about/dei-resource-center/). The American Association of Nurse Practitioners (AANP) offers similar resources and groups them according to the domains of the organization: practice, continuing education, advocacy, research, and leadership (https://www.aanp.org/diversity-equity-and-inclusion).

Ultimately, whatever strategies and resources are employed to develop a useable framework for implicit bias training, successful implementation is dependent on consistency in training. One-time training sessions do not offset deep cultural associations and stereotypes; in fact, they can be harmful, suggesting that a 30-min module remedies implicit bias. Brief training sessions may be well-intentioned and designed to promote awareness, but most often does not lead to a sustained behavioral change [37]. While incorporating implicit bias training is a critical step, it certainly is not the end goal. To effect change, educators and organizational leadership must be aligned in their strategic focus and demonstrate commitment to inclusion through an integrated process invested in continuity.

### Strengths and limitations

A strength of this study is the use of a cross-sectional survey to help derive an understanding of implicit bias instruction in PA and joint PA/NP postgraduate fellowship/residency training. Because this study describes attitudes related to implicit bias training in postgraduate PA and NP education, it establishes important implications for further research. A limitation of our study was the survey response rate of (41%). The low response rate may have led to non-response bias, and respondent feedback was not representative of all postgraduate PA and joint PA/NP postgraduate programs affiliated with APPAP. Therefore, generalizability of our findings is limited, as the percentage of respondents were less than a majority of those in our sample population. It is worth mentioning that previous research has demonstrated that web-based surveys are not without challenges, given there is an 11% lower response rate than other survey modes [38]. Another limitation is the wide range of classroom and clinical experiences across institutions and between countries, making it difficult to accurately review and evaluate the growing concern for implicit bias training in health care programs. Lastly, using a Likert scale to evaluate attitudes about implicit bias can be associated with response bias.

## Conclusion

To our knowledge, this is the first study to investigate the current state of implicit bias training in PA and joint PA/NP postgraduate residency/fellowship programs. Our findings unmask the key barriers to offering implicit bias training in some PA and joint PA/NP postgraduate education programs and the need for a focused framework to help guide the development and implementation of implicit bias training in these programs.

### Areas of future research

An area of future inquiry is to examine the effectiveness of current training offerings and whether postgraduate PA and NP trainees who have completed implicit bias training regularly engage in bias-reducing and bias-managing strategies [39]. Another area of exploration is the training frequency, duration, and perceived efficacy of implicit bias instruction among postgraduate PA and NP trainees.

## Data Availability

A summary of the datasets generated and/or analyzed during the current study are not publicly available but are available from the corresponding author on reasonable request in accordance with the IRB approved protocol.
